# Community Engagement and Human-Centered Design: Lessons From HEARS in Inclusive Recruitment of Older Adults

**DOI:** 10.1093/geroni/igab046.1822

**Published:** 2021-12-17

**Authors:** Carrie Nieman, Hae-Ra Han, Nicole Marrone, Jonathan Suen, Sarah Szanton, Jami Trumbo, Frank Lin

**Affiliations:** 1 Johns Hopkins University School of Medicine, Baltimore, Maryland, United States; 2 Johns Hopkins School of Nursing, Baltimore, Maryland, United States; 3 University of Arizona, Tucson, Arizona, United States; 4 Johns Hopkins University School of Nursing, Baltimore, Maryland, United States; 5 Johns Hopkins University, Baltimore, Maryland, United States; 6 Cochlear Center for Hearing & Public Health, Baltimore, Maryland, United States; 7 Johns Hopkins University, Johns Hopkins University, Maryland, United States

## Abstract

Within hearing care, significant disparities persist despite the highly prevalent nature of age-related hearing loss and relatively few trials include representation of racial/ethnic minorities. HEARS is an affordable, accessible hearing care intervention delivered by older adult peer mentors. The HEARS randomized controlled trial (NCT03442296) is a community-engaged RCT with an embedded human-centered design practitioner. Recruitment efforts occurred over 18 months in partnership with 13 affordable housing and social centers. The cohort (n=151) includes 43% (n=65) who self-identify as African American and 63.6% (n=96) with <$25,000 annual household income. The cohort represents the largest to-date of African American and low-income older adults with hearing loss. Recruitment efforts entailed 470.5 staff hours and $4,917.26 in supplies, equating to 1.4 hours and $14.13 per 1 individual screened and 3.1 hours and $32.56 for 1 participant randomized. Community-engaged research, partnered with human-centered design, may offer critical approaches to increasing representation within behavioral intervention trials.

